# Toll-like receptors 1, 2, 4 and 6 in esophageal epithelium, Barrett's esophagus, dysplasia and adenocarcinoma

**DOI:** 10.18632/oncotarget.8151

**Published:** 2016-03-17

**Authors:** Heikki Huhta, Olli Helminen, Petri P. Lehenkari, Juha Saarnio, Tuomo J. Karttunen, Joonas H. Kauppila

**Affiliations:** ^1^ Department of Pathology, University of Oulu, 90014 Oulu, Finland; ^2^ Department of Surgery, University of Oulu, 90014 Oulu, Finland; ^3^ Department of Anatomy and Cell biology, University of Oulu, 90014 Oulu, Finland; ^4^ Medical Research Center Oulu, 90014 Oulu, Finland; ^5^ Oulu University Hospital, 90029 Oulu, Finland

**Keywords:** Toll-like receptor 1, Toll-like receptor 2, Toll-like receptor 4, Toll-like receptor 6, esophageal adenocarcinoma

## Abstract

**Background:**

Toll-like receptors (TLRs) recognize microbial and endogenous ligands and have already shown to play a role in esophageal cancer. In this study, we evaluated especially TLRs that sense bacterial cell wall components in Barrett's esophagus, dysplasia and esophageal adenocarcinoma.

**Methods:**

TLRs 1, 2, 4 and 6 were stained immunohistochemically and assessed in esophageal specimens from patients with esophageal dysplasia (*n* = 30) or adenocarcinoma (*n* = 99). Structures and lesions were evaluated including normal esophagus (*n* = 88), gastric (*n* = 67) or intestinal metaplasia (*n* = 51) without dysplasia, and low-grade (*n* = 42) or high-grade dysplasia (*n* = 37), and esophageal adenocarcinoma (*n* = 99).

**Results:**

We found TLR1, TLR2, TLR4 and TLR6 expression in all lesions. TLR expression increased in Barrett's mucosa and dysplasia. There was profound increase of TLR expression from gastric- to intestinal-type columnar epithelium. In cancers, high nuclear and cytoplasmic staining of TLR4 associated with metastatic disease and poor prognosis.

**Conclusions:**

TLR1, TLR2, TLR4 and TLR6 are upregulated during malignant changes of esophageal columnar epithelium. Increased TLR4 expression associates with advanced stage and poor prognosis in esophageal adenocarcinoma.

## INTRODUCTION

Toll-like receptors (TLRs) are innate immune receptors with unique antigen-recognition domains. Toll-like receptor 4, for example recognizes lipopolysaccharide and TLRs 1, 2 and 6 form heterodimers to recognize different kinds of lipopeptides, which are components of bacterial cell wall [1]. Bacteria-recognizing TLRs are found, not only in immune cells, but also in epithelial cells and fibroblasts. Epithelial cells sense luminal pathogens via TLRs and activate immune cells and consequent inflammation [2].

Bacterial infection affects carcinogenesis by altering cytokine and chemokine expression. These stimulate inflammation, angiogenesis and metastasis [3]. The best characterized pathway is correlation between gastric cancer and *Helicobacter pylori* infection [4], where aberrant TLR expression is involved [5]. Esophageal microbiome shows characteristic features in Barrett's esophagus and esophageal adenocarcinoma, but their actual pathogenetic significance is not known [6].

In esophageal epithelium, TLR9 expression increases during premalignant and malignant changes [7–9] and TLR9 activation stimulates invasion in esophageal adenocarcinoma cells [10]. Epithelial TLR5 expression increases along with development esophageal columnar dysplasia and is a marker of dysplasia [11]. No published information on TLRs 1, 2, 4 or 6 in esophageal dysplasia or adenocarcinoma could be found [12].

The aim of this study was to assess the expression of TLRs 1, 2, 4 or 6 in different stages of esophageal metaplasia-dysplasia-adenocarcinoma-sequence.

## RESULTS

### Expression of TLRs 1, 2, 4 and 6 in esophageal squamous epithelium, in Barrett's esophagus, dysplasia and cancer

TLRs 1, 2, 4 and 6 were all expressed in normal and metaplastic esophagus. The expression of all of these TLRs was the lowest in normal esophageal squamous epithelium and increased towards to high-grade dysplasia. In cancer, the expression was the most variable. Expression of TLRs was mainly cytoplasmic. The percentage of cytoplasmic staining was 100% in nearly all lesions in all of the TLRs. Histoscores for the different TLRs summarised in Table 1.

**Table 1:** Baseline characteristics of TLR1, 2, 4 and 6 expression in normal esophageal squamous epithelium and in different esophageal lesions

TLR1	histoscore mean	histoscore median	histoscore IQR	statistical significance	nuclei mean	median	IQR	statistical significance
Normal epithelium	106	100	10		57	53	65	bcdef
Gastric metaplasia	143	135	100	a	16	5	28	def
Intestinal metaplasia	194	200	63	ab	18	5	35	ef
Low-grade dysplasia	222	200	100	abcf	10	0	15	f
High-grade dysplasia	243	250	100	abcf	5.7	0	5	f
Adenocarcinoma	189	200	150	ab	4.0	0	0	
TLR2								
Normal epithelium	108	100	10		-			
Gastric metaplasia	137	150	118	a	-			
Intestinal metaplasia	158	170	100	ab	-			
Low-grade dysplasia	185	200	74	ab	-			
High-grade dysplasia	241	200	200	abc	-			
Adenocarcinoma	202	200	100	abc	-			
TLR4								
Normal epithelium	105	100	1		60	85	80	bcdef
Gastric metaplasia	160	150	100	a	39	30	83	f
Intestinal metaplasia	244	250	100	ab	43	38	85	f
Low-grade dysplasia	218	225	75	ab	35	0	95	
High-grade dysplasia	247	250	100	ab	31	0	85	
Adenocarcinoma	234	250	100	ab	31	0	95	
TLR6								
Normal epithelium	119	100	44		-			
Gastric metaplasia	151	150	100	a	-			
Intestinal metaplasia	211	200	50	ab	-			
Low-grade dysplasia	242	250	100	abc	-			
High-grade dysplasia	245	300	100	abcf	-			
Adenocarcinoma	219	250	150	ab	-			

Intensity was assessed with a four point scale from negative (0) to strong intensity (3). The extent of the staining was expressed as percentage of positive cells and positive cell nuclei (0–100%). Histoscore is counted by multiplying intensity with the percentage of positive cells (0–300) Values are presented as mean, median and interquartile range (IQR). Statistically significant differences are shown for histoscores and nuclear TLR expression. Letters are placed to indicate the lesion with higher TLR expression.

**a** compared to normal epithelium, *p* < 0.05.

**b** compared to gastric metaplasia, *p* < 0.05.

**c** compared to intestinal metaplasia, *p* < 0.05.

**d** compared to low-grade dysplasia, *p* < 0.05.

**e** compared to high-grade dysplasia, *p* < 0.05.

**f** compared to adenocarcinoma, *p* < 0.05.

**Figure 1:**
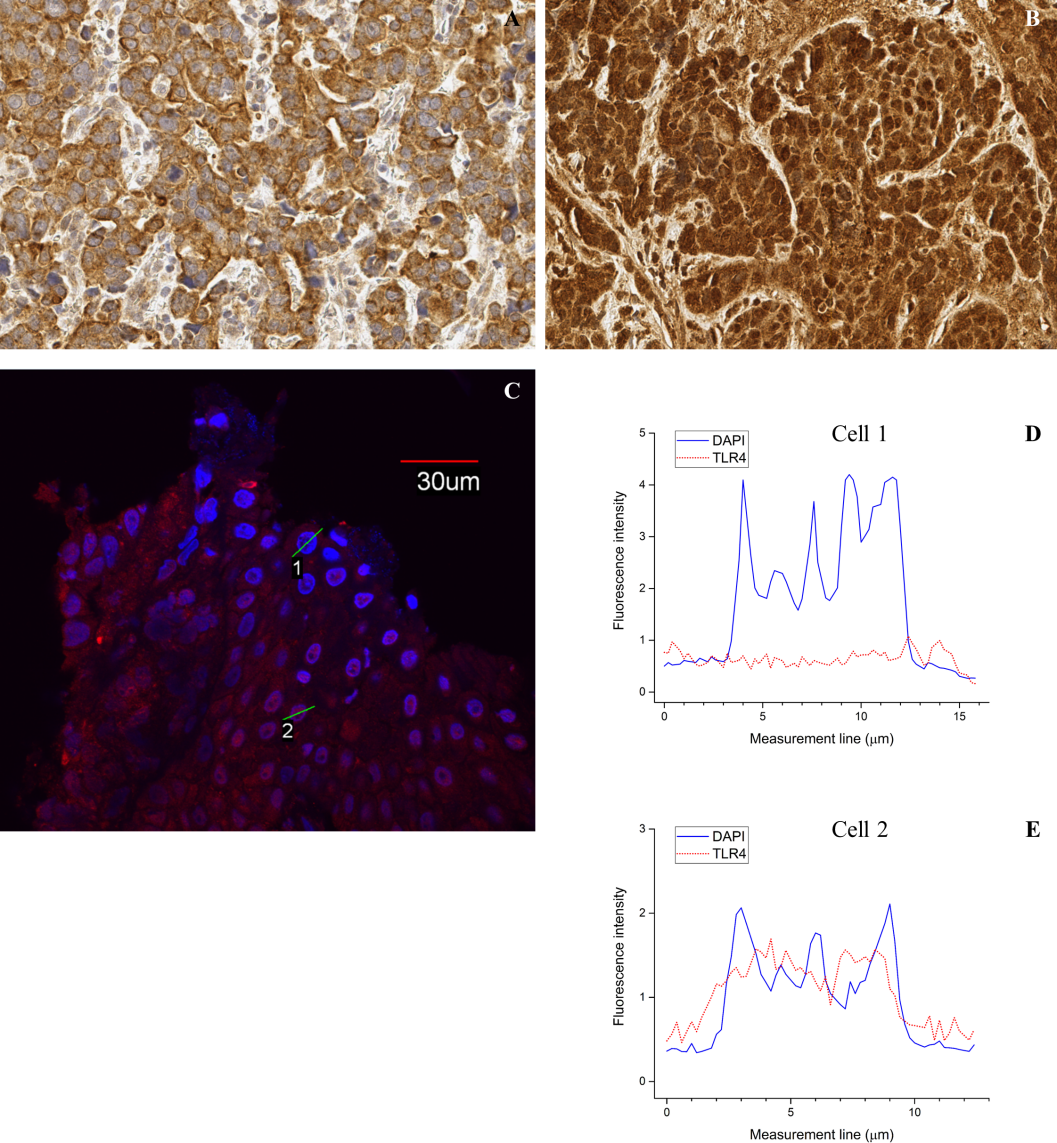
Examples of nuclear TLR4 expression Immunohistochemical staining showing negative (**A**) and positive (**B**) nuclear TLR4 staining. Immunofluorescence confirming variable nuclear expression with examples of both TLR4 (red label) negative (cell 1) and positive (cell 2) nuclei in the same carcinoma sample (**C**). Nuclei are marked with DNA specific DAPI staining (blue). Corresponding intensity profiles of TLR (**D**, **E**) of carcinoma cells with TLR4 positive cytoplasm but negative nucleus (see figure C, Cell 1) and both positive cytoplasm and nucleus (see figure C, Cell 2). Solid line shows the intensity of DAPI and dotted line the intensity of TLR4. Magnification 40x (IHC) and 60x (IF) were used.

Interestingly, TLR1 (14%; 14/99) and TLR4 (33%; 33/99) showed nuclear staining in esophageal adenocarcinoma. Freely available NucPred analysis was done to predict the probability of translocation of these proteins to the nucleus [13]. NucPred score (range 0–1.0) was 0.61 for TLR1 and 0.43 for TLR4, meaning that it is somewhat probable that these proteins translocate to nucleus. By using immunofluorescence analysis, we confirmed the nuclear localization of TLR4 as shown in Figure 1. We could not confirm nuclear localization for TLR1 (data not shown).

The TLR1 histoscore was increased in both types of metaplasia when compared to normal epithelium. The expression of TLR1 was the highest in high-grade dysplasia and similar in low-grade dysplasia and intestinal metaplasia. Nuclear expression of TLR1 became more infrequent during preneoplastic and neoplastic changes, when compared to normal epithelium. The expression of TLR2 was slightly, but significantly increased from normal epithelium towards adenocarcinoma. Toll-like receptor 4 expression was the lowest in normal epithelium and gastric metaplasia. The expression of TLR4 was similar between intestinal metaplasia, low- and high-grade dysplasia and adenocarcinoma. TLR6 had similar pattern of expression to other TLRs. TLR6 intensity increased from normal epithelium and gastric metaplasia towards intestinal metaplasia and high-grade dysplasia, which had clearly the highest TLR intensity.

**Figure 2:**
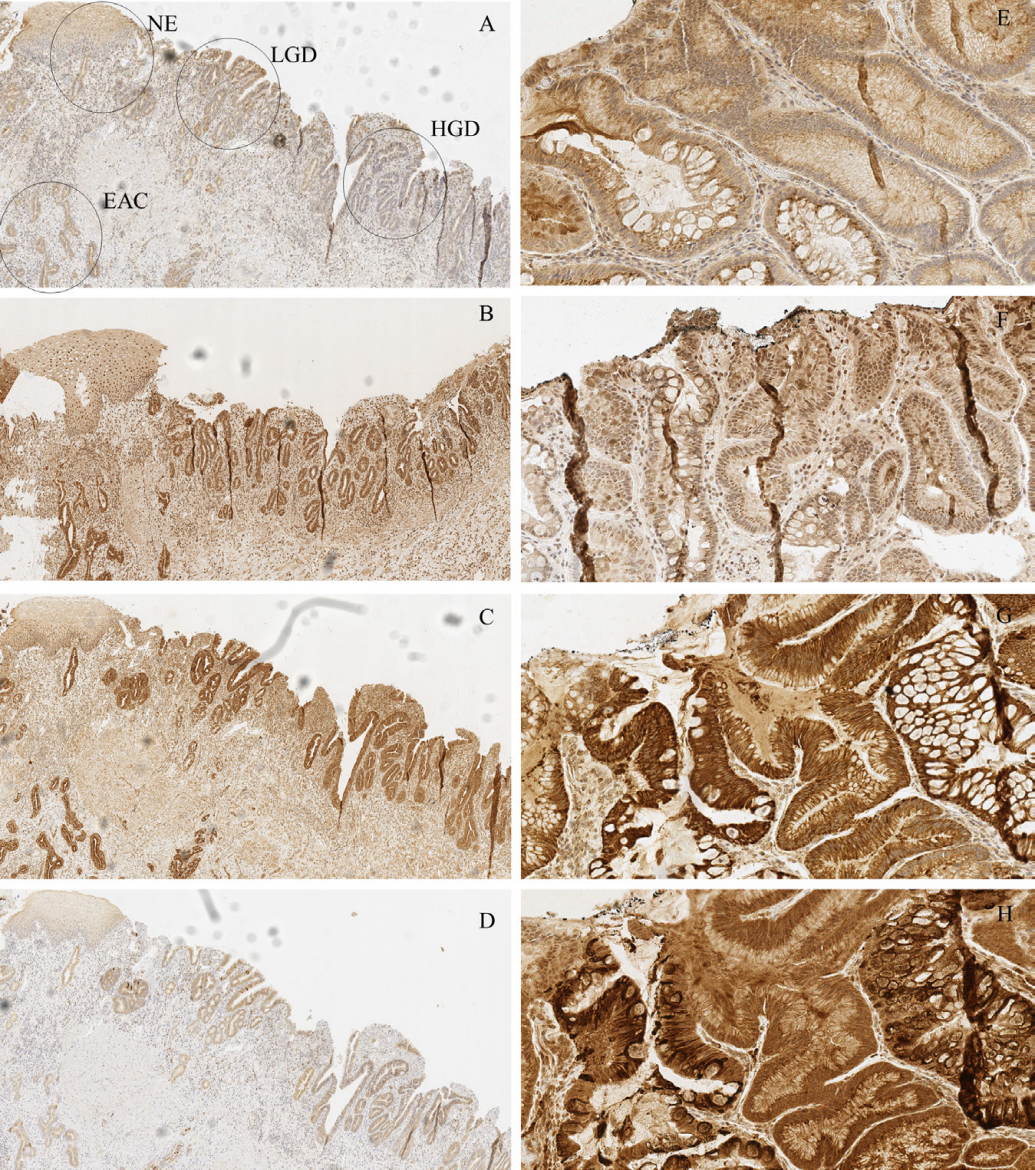
Examples of typical expression patterns of TLR1 (A, E), TLR2 (B, F), TLR4 (C, G) and TLR6 (D, H) (**A–D**) represent the same sample with normal epithelium (NE), low-grade dysplasia (LGD), high grade dysplasia (HGD) and esophageal adenocarcinoma (EAC) marked in the figure A. Gradual increase is found through normal epithelium – metaplasia – dysplasia sequence. E-H show intestinal type metaplasia (left) and gastric type metaplasia (right). Gastric metaplasia presented a strong polarized staining to the basal cytoplasm in TLR 1, 4 and 6 stainings. TLR1 and TLR2 show basal polarization in intestinal metaplasia, whereas TLR4 and TLR6 are expressed more diffusely. Expression pattern of all studied TLRs in adenocarcinoma is diffuse extending homogenously throughout the cell cytoplasm with no apparent basal polarization. Magnifications 6× and 20× were used.

Generally the expression of all examined TLRs increased from normal epithelium towards high-grade dysplasia. The most profound increase in the TLR staining was observed in transition from gastric metaplasia to intestinal-type metaplasia. The examples of immunohistochemical stainings with different TLRs are shown in Figure 2.

### Relation between TLRs, clinicopathological variables and cancer survival

**Figure 3:**
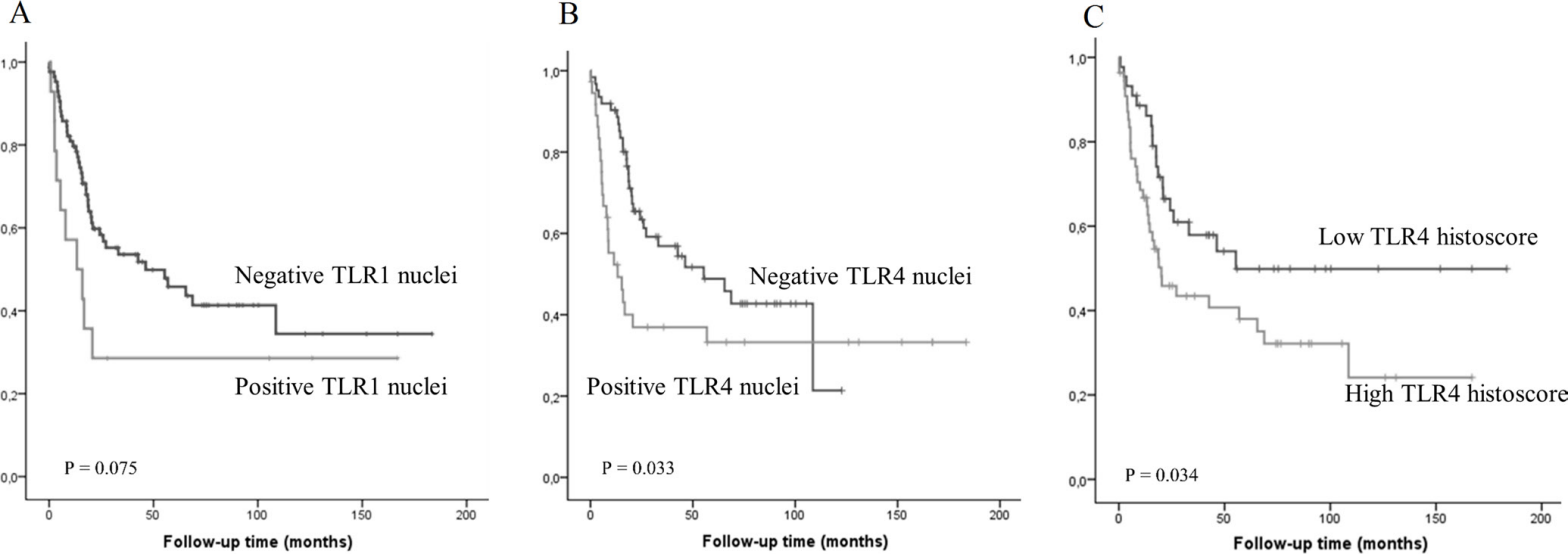
Kaplan-Meier curve showing esophageal adenocarcinoma survival stratified by nuclear TLR1 (A) or TLR4 (B) expression and TLR4 histoscore (C)

Presence of nuclear expression of TLR1 correlated with distant metastases (*P* < 0.001). However, the nuclear TLR1 expression could not be confirmed. High TLR4 expression associated to high T-class (*P* < 0.01) and nuclear expression of TLR4 also correlated with distant metastases (*P* < 0.001). TLR4 histoscore and nuclear expression predicted survival in univariate (*P* < 0.05, Figure 3), but not in multivariate analysis (data not shown). Nuclear TLR1 expression showed survival trend (*P* = 0.075, Figure 3). TLR2 and 6 expression in cancer tissue was not associated to clinicopathological parameters or prognosis (data not shown). Table 2 summarizes the relation between clinicopathological variables, nuclear expressions and histoscores of TLR1 and 4.

**Table 2:** TLR1 and 4 nuclear expressions and histoscores compared to clinicopathological variables in esophageal adenocarcinoma

Variable	n/N (%)	TLR1 nuclei n (%)			TLR4 nuclei n (%)			TLR1 histoscore n (%)			TLR4 histoscore n (%)		
		Absent	Present	*p*	Absent	Present	*p*	Weak	Strong	*p*	Weak	Strong	*P*
pT													
T1–2	29/94 (31)	25 (31)	4 (31)	0.995	22 (36)	7 (21)	0.137	11 (27)	18 (34)	0.458	18 (43)	11 (21)	**0.024**
T3–4	65/94 (69)	56 (69)	9 (69)		39 (64)	26 (79)		30 (37)	35 (66)		24 (57)	41 (79)	
Lymph nodes													
negative	35/94 (37)	33 (41)	2 (15)	0.079	27 (44)	8 (24)	0.055	11 (27)	24 (45)	0.066	18 (43)	17 (33)	0.311
positive	59/94 (63)	48 (59)	11 (85)		34 (56)	25 (76)		30 (73)	29 (55)		24 (57)	35 (67)	
Organ metastases													
negative	63/94 (67)	60 (74)	3 (23)	**< 0.001**	51 (84)	12 (36)	**< 0.001**	26 (63)	37 (70)	0.513	31 (74)	32 (62)	0.208
positive	31/94 (33)	21 (26)	10 (77)		10 (16)	21 (64)		15 (37)	16 (30)		11 (26)	20 (39)	
Grade													
1	29/93 (31)	26 (33)	3 (23)	0.864	22 (37)	7 (21)	0.136	13 (33)	16 (30)	0.516	13 (32)	16 (31)	0.497
2	22/93 (24)	19 (24)	3 (23)		16 (27)	6 (18)		7 (18)	15 (28)		12 (29)	10 (19)	
3	42/93 (45)	35 (44)	7 (54)		22 (37)	20 (61)		20 (50)	22 (42)		16 (39)	26 (50)	
Stage													
I	13/94 (14)	13 (16)	0 (0)	**0.003**	11 (18)	2 (6)	**< 0.001**	4 (10)	9 (17)	0.232	9 (21)	4 (8)	0.162
II	37/94 (39)	34 (42)	3 (23)		31 (51)	6 (18)		19 (46)	18 (34)		17 (41)	20 (39)	
III	13/94 (14)	13 (16)	0 (0)		9 (15)	4 (12)		3 (7)	10 (19)		6 (14)	7 (14)	
IV	31/94 (33)	29 (26)	10 (77)		10 (16)	21 (64)		15 (37)	16 (30)		10 (24)	21 (40)	
Tumor resection													
unresectable	28/99 (28)	19 (22)	9 (64)	**0.001**	3 (5)	25 (68)	**< 0.001**	12 (28)	16 (29)	0.942	8 (18)	20 (36)	**0.046**
resected	71/99 (72)	66 (78)	5 (39)		59 (95)	12 (32)		31 (72)	40 (71)		36 (82)	35 (64)	

Significant *p*-values are shown in bold.

## DISCUSSION

We demonstrate widespread expression of TLRs 1, 2, 4 and 6 in normal esophageal squamous epithelium, columnar metaplasia and dysplasia of Barrett's esophagus, as well as esophageal adenocarcinoma. Expression of all of these TLR types showed general pattern of stepwise increase in metaplasia, dysplasia and adenocarcinoma. By American definition, Barrett's esophagus is defined as columnar-lined esophagus, with intestinal-type goblet cells in the epithelium. Some studies have shown increased risk of cancer in intestinal metaplasia compared to gastric metaplasia [14]. The largest incremental increase in TLR expression was observed in between gastric-type and intestinal-type metaplasia, suggesting major inflammatory changes between these two epithelial types. Interestingly, TLR4 expression in the cytoplasm and the presence of nuclear TLR4 expression correlated to distant metastases and poor prognosis.

Based on our results, TLR1/2/6-network is upregulated in Barrett's metaplasia, dysplasia, and cancer. Activation of these receptors induces inflammatory reactions via NF-kappaB [1]. In cancers, ligands of these TLRs have been shown to stimulate inflammatory cytokines, but also to induce tumor regression [15]. In agreement with present findings, earlier studies have reported TLRs 1, 2 and 6 to be expressed in normal esophagus. TLR2 is known to upregulate beta-defensin 2 upon activation in esophageal cells [16]. Upregulation of TLR1/2/6-network in Barrett's metaplasia, dysplasia, and cancer could mark improved recognition of bacteria by precancerous metaplastic and dysplastic cells leading to inflammation, which is one of the hallmarks of cancer [3]. Upregulation in the TLR1/2/6 network could possibly increase the recognition of fungal organisms. Proton-pump inhibitor treatment increases the risk esophageal Candida colonization [17]. This might contribute to the proinflammatory cascade in Barrett's esophagus. Expression levels of TLR1 and TLR6 showed decrease in carcinomas as compared with premalignant epithelium. Variation of expression was high in carcinomas suggesting that increased aberration of cellular regulatory mechanisms could be a reason leading to downregulation in some cases. Another potential mechanism is that invasive cells in carcinomas do not have contact with lumen and therefore are less in contact with luminal TLR ligands. This could modify the expression of TLRs.

Cytoplasmic TLR4 expression increased towards dysplasia and cancer. Previous studies have shown TLR4 expression in various cancer types. [18–23] TLR4 activation has been linked to increased invasion and nodal metastasis in breast cancer, as well as induction of tumor growth in ovarian cancer [20, 21]. TLR4 knockdown attenuates tumor growth in lung cancer [19]. Supporting role of TLR4 activation in pathogenesis of esophageal inflammation and carcinoma, TLR4 activation has been shown to induce Interleukin-8 and NF-kB in esophageal epithelial cells and, more pronouncedly so, in Barrett's esophagus. TLR4 activation also increased cyclo-oxygenase 2 expression in Barrett's esophagus [24]. These results suggest that TLR4 activation is involved in pathogenesis of esophageal adenocarcinoma, and that increased cyclo-oxygenase 2 activation is a mediator of this activation similar to gastric cancer [25].

Although proportion of epithelial cell nuclei with TLR1 and TLR4 expression decreased towards malignancy, the presence of nuclear staining for both TLR1 in (12%) and TLR4 (33%) was common in esophageal adenocarcinomas. Interestingly, for TLR4 the nuclear staining correlated to distant metastasis and poor prognosis.

The mechanism of nuclear translocation of TLR4 and the correlation with prognosis remain speculative. TLR4 contains several sequences indicating nuclear localization [13]. Alternatively, nuclear translocation might be related with carrier proteins, but no such proteins have been identified. By using transcription factor sequence identification analysis program (www.transcriptionfactor.org) we could not find potential transcription factor sequences in TLR4 [26]. However, we think that translocation of these membrane-bound TLRs to nucleus is due to increased amount of these proteins and related signaling activity. Low nuclear expression of TLR4 has been previously linked to development of laryngeal squamous cell carcinoma [27]. However, this finding was not confirmed by other techniques.

In conclusion, upregulation of bacterial and fungal component-sensing TLRs is present in esophageal metaplasia-dysplasia-adenocarcinoma sequence. TLR expression is greatly increased in transition from gastric metaplasia to intestinal metaplasia, suggesting changes in innate immune activation between these two conditions. High cytoplasmic expression of TLR4 and presence of nuclear expression of TLR4 associate with advanced stage and poor prognosis in esophageal adenocarcinoma

## MATERIALS AND METHODS

### Patients

Paraffin-embedded, archival specimens of esophageal adenocarcinoma or esophageal dysplasia were obtained from the Department of Pathology, Oulu University Hospital, between the years 1987–2013. The final series consisted of 99 patients with esophageal adenocarcinoma, 10 with high-grade dysplasia, and 20 with low-grade dysplasia as the most advanced lesion. The material has been earlier described elsewhere [11]. The median age of the cancer patients was 64 years (range 43–90). The median follow-up time was 36 months (range 0–288 months) for the surviving patients. The patient survival data was acquired from Statistics Finland, and the other relevant data was acquired from the patient records (Table 3). We could not retrieve full clinical data from 6 of the patients and survival data from 5 of the cancer patients.

**Table 3:** Baseline characteristics of the patients with esophageal adenocarcinoma (EAC), high-grade dysplasia (HGD) and low-grade dysplasia (LGD)

Patient clinical data	EAC *N* = 99		HGD *N* = 10		LGD *N* = 20	
Age at diagnosis	n/N	%	n/N	%	n/N	%
< 60 yrs	34/99	34	5/10	50	4/20	20
60–65 yrs	21/99	21	3/10	30	3/20	15
> 65 yrs	43/99	43	2/10	20	13/20	65
Sex						
Male	82/99	83	10/10	100	13/20	65
Female	17/99	17	0/10	0	7/20	35
Tumor grade						
1	29/92	32				
2	22/92	24				
3	41/92	45				
T-classification						
I	13/93	14				
II	14/93	15				
III	51/93	55				
IV	15/93	16				
Lymph nodes						
Negative	35/93	62				
Positive	58/93	38				
Distal metastases						
Negative	63/93	68				
Positive	30/93	32				
Tumor stage						
I	13/93	14				
II	37/93	40				
III	13/93	14				
IV	30/93	32				

Total of 99 patients with EAC, 10 patients with HGD and 20 with LGD are presented in the Table. Full clinical data could not be obtained from 7 patients.

The use of patient samples and the data inquiry were approved by the Oulu University Hospital Ethics Committee. The need to obtain a written or oral consent from the patients for using the samples in research was waived by the Finnish National Authority for Medicolegal Affairs (VALVIRA, Dnro 10832/06.01.03.01/2014).

### Immunohistochemistry

Immunohistochemistry was performed on the tissue block sections, which were first selected by expert gastrointestinal pathologist, on the basis of hematoxylin and eosin-staining, to be representative for the tumor mass in the resected specimen. TLR immunostaining was performed with a commercial monoclonal antibody (IMG-5012, rabbit IgG1, Clone N/A for TLR1, MAB0066, mouse IgG1, lot number 0062201B-09 for TLR2, H00007099-M02, mouse IgG2a kappa, lot number 11277-3B6 for TLR4 and PAB3555, polyclonal rabbit IgG1, lot number SH030317H for TLR6 Imgenex, San Diego, CA and Abnova Corporation) at dilutions of 1:75 (TLR1), 1:75 (TLR2), 1:1000 (TLR4) and 1:750 (TLR6). For immunohistochemical detection of the antibody reaction, we used the Dako Envision kit (Dako, Copenhagen, Denmark) with a high temperature antigen retrieval in Tris-EDTA (for Ki67, TLR9) buffer for 15 minutes. Diaminiobenzidine (Dako basic DAB-kit) was used as a chromogen. All staining was done with Dako Autostainer (Dako, Copenhagen, Denmark).

We validated the immunohistochemical analysis through positive and two series of negative controls (omitting the primary antibody and by replacing primary antibody with the mouse primary antibody isotype control). Lymphocytes of the lymph nodes in the sample material were used as an internal positive control for TLR staining. To assess the effect of paraffin block age on the preservation of the studied antigens we compared the TLR staining intensities in normal esophageal epithelium between old and new blocks (grouped by the median age of the blocks). No significant differences were found indicating that age of tissue blocks does not significantly affect staining intensity

### Immunofluorescence

Formalin-fixed and paraffin-embedded esophageal sections were deparaffinized followed by treatment with citrate buffer in pH 6. Nonspecific staining was blocked by treatment with 1% bovine serum albumin for 20 min and 150 ul of normal gout serum. Incubation with primary antibodies for 60 min (TLR4) and overnight (TLR1) at room temperature was then performed. The primary antibodies used are described in the immunohistochemistry section. After several washes, Alexa Fluor 568 or Alexa Fluor 594 conjugated to goat anti-rabbitt (TLR1) and goat anti-mouse IgG (TLR4) (Life Technologies) was applied at appropriate dilutions and incubated for 30 min at room temperature. DNA specific DAPI staining was applied. Samples were mounted with H-1200 (Vector, Gdynia, Poland) and examined by using a Olympus FluoView FV1000 confocal microscope (Olympus corp, Tokyo, Japan). Used objective was 60x/1.35. Excitation and detection wavelengths for DAPI were 405 nm and 430– 470 nm, and 568 nm (TLR4) with long pass 560 nm for Alexa fluor 568 and 594.

### Assessment of TLR expression

The histological sample slides were digitized using Aperio AT2 Console, Leica Biosystems Imaging Inc, Nussloch, Germany, Different lesions in the specimens were identified and marked by an expert gastrointestinal pathologist (T.J.K.). TLR immunoreactivity was analyzed by two independent researchers who were blinded from the clinical data, using method described earlier [11]. We assessed the intensity of staining (0–3) and the percentage of staining (0–100) in different lesions. Mean values of two independent estimates were used if there was no difference over 1 in the intensity or over 30% in the percentage. If the difference was more extensive, consensus was reached after re-evaluation with a third researcher. Re-evaluation was however not needed in any of the samples, indicating a good reproducibility. Mean intensity and mean percentage was then multiplied together to obtain a histoscore (0–300). Histoscore was dichotomized into equally sized groups by the median value of each TLR histoscore. Percentage of cells showing membranous and nuclear staining was also assessed. After the analysis, the samples were dichotomized by nuclear staining, the staining being either “0 = absent” or “1 = present”.

Intensity profiles of TLR4 and DAPI were acquired from immunofluorescence sample by analyzing the detectable fluorescence with 0.2 μm interval horizontally through the selected cells. Olympus FluoView FV1000 software was used in the analysis.

### Statistical analysis

We used IBM SPSS Statistics 22.0 (IBM corp., Armonk, NY) for statistical analyses. To compare TLRs expression between different lesions we used Kruskall-Wallis due to skewed distributions. The chi-square-test was used to calculate statistically significant differences between prognostic and clinicopathologic variables. Life tables were calculated according to the Kaplan-Meier method, and the survival curves were compared using the log-rank test. Cox proportional hazards model with backward selection was used for multivariate analysis with following covariates: Age, gender, *T*-stage, *N*-stage, *M*-stage and grade of differentiation.
